# Galectin-1 Expression Is Associated with the Response and Survival Following Preoperative Chemoradiotherapy in Locally Advanced Esophageal Squamous Cell Carcinoma

**DOI:** 10.3390/cancers13133147

**Published:** 2021-06-23

**Authors:** Shau-Hsuan Li, Yen-Hao Chen, Hung-I Lu, Chien-Ming Lo, Chao-Cheng Huang, Yu-Ming Wang, Eng-Yen Huang

**Affiliations:** 1 Department of Hematology-Oncology, Kaohsiung Chang Gung Memorial Hospital, Chang Gung University College of Medicine, Kaohsiung 833, Taiwan; lee0624@cgmh.org.tw (S.-H.L.); alex2999@cgmh.org.tw (Y.-H.C.); 2 Department of Thoracic & Cardiovascular Surgery, Kaohsiung Chang Gung Memorial Hospital, Chang Gung University College of Medicine, Kaohsiung 833, Taiwan; luhungi@cgmh.org.tw (H.-I.L.); t123207424@cgmh.org.tw (C.-M.L.); 3 Department of Pathology, Kaohsiung Chang Gung Memorial Hospital, Chang Gung University College of Medicine, Kaohsiung 833, Taiwan; huangcc@cgmh.org.tw; 4 Department of Radiation Oncology, Kaohsiung Chang Gung Memorial Hospital, Chang Gung University College of Medicine, Kaohsiung 833, Taiwan; scorpion@cgmh.org.tw

**Keywords:** esophageal cancer, squamous cell carcinoma, Galectin-1, chemoradiotherapy, pathological complete response

## Abstract

**Simple Summary:**

Galectin-1 has been found to be involved in therapeutic resistance in a variety of cancers. However, the prognostic significance of galectin-1 expression in patients with locally advanced esophageal squamous cell carcinoma (ESCC) treated with chemoradiotherapy remains unknown. Immunohistochemically, we observed that galectin-1 overexpression in pretreatment biopsied specimens was significantly associated with a lower pathological complete response rate, worse overall survival and disease-free survival in 93 patients with locally advanced ESCC receiving preoperative chemoradiotherapy. Our findings suggest that galectin-1 may be a potential therapeutic target for patients with ESCC treated with preoperative chemoradiotherapy.

**Abstract:**

The galectin-1 has been found to be involved in poor outcomes after treatment of a variety of cancers. To the best of our knowledge, however, the significance of galectin-1 expression in the sensitivity to chemoradiotherapy (CCRT) of patients with locally advanced esophageal squamous cell carcinoma (ESCC) remains unclear. Expression levels of galectin-1 were evaluated by immunohistochemistry and correlated with the treatment outcome in 93 patients with locally advanced ESCC who received preoperative CCRT between 1999 and 2012. Galectin-1 expression was significantly associated with the pathological complete response (pCR). The pCR rates were 36.1% and 13.0% (*p* = 0.01) in patients with low and high galectin-1 expression, respectively. Univariate analyses revealed that galectin-1 overexpression, clinical 7th American Joint Committee on Cancer (AJCC) stage III and a positive surgical margin were significant factors of worse overall survival and disease-free survival. In multivariate analyses, galectin-1 overexpression and a positive surgical margin represented the independent adverse prognosticators. Therefore, galectin-1 expression both affects the pCR and survival in patients with locally advanced ESCC receiving preoperative CCRT. Our results suggest that galectin-1 may be a potentially therapeutic target for patients with ESCC treated with preoperative CCRT.

## 1. Introduction

The treatment outcomes of patients with locally advanced esophageal squamous cell carcinoma (ESCC) undergoing surgery alone is poor [1,2]. Therefore, concurrent chemoradiotherapy (CCRT) followed by esophagectomy has been suggested for these patients in order to improve the outcome [3]. However, the necessity for an esophagectomy after CCRT remains unclear. In fact, phase III clinical trials [3,4] demonstrated that an esophagectomy may be unnecessary for those patients who respond well to CCRT. There are 20–40% patients who can achieve a pathological complete response (pCR) following preoperative CCRT. Therefore, these patients with pCR have a significantly improved survival [5,6,7]. However, there is still at least one half of patients without pCR after CCRT. Therefore, it is important to explore the biomarkers involved in the resistance to CCRT and identify patients with a good response to CCRT to avoid potential perioperative complications.

Galectins are members of a lectin family and an affinity for ß-galactose-containing oligosaccharides [8]. There are 15 different galectins found. Galectin-1 is widely expressed in many tissues and has many cellular functions, such as proliferation [9], cellular signaling, adhesion, migration [10] and apoptosis [11]. In the tumor microenvironment, galectin-1 has been shown to be involved in immune suppression [11], tumor angiogenesis [12] and metastasis [13]. Previous studies showed that galectin-1 was regulated by hypoxia [14,15,16], and tumor hypoxia is well-known to be related to resistance to radiotherapy or chemotherapy [17]. Galectin-1 is involved in the resistance to radiotherapy or chemotherapy in several types of cancer including squamous cell carcinoma of the uterine cervix [18], hepatocellular carcinoma [19], glioblastoma [20,21], epithelial ovarian cancer [22] and lung carcinoma [23,24]. However, the significance of galectin-1 expression to the sensitivity of preoperative CCRT in patients with locally advanced ESCC remains unclear. The aim of this study was to investigate whether galectin-1 expression can predict the sensitivity of CCRT in patients with locally advanced ESCC. Thus, we investigated the galectin-1 expression by immunohistochemistry (IHC) and demonstrated its role in 93 patients with locally advanced ESCC treated with preoperative CCRT.

## 2. Materials and Methods

### 2.1. Patient Population

This study was approved by the Institutional Review Board of Chang Gung Memorial Hospital (IRB number: 202100009B0), Kaohsiung, Taiwan, R.O.C. From January 1999 to December 2012, patients who had a diagnosis of esophageal cancer and received treatment at Kaohsiung Chang Gung Memorial Hospital were reviewed retrospectively. During this period, 769 patients diagnosed with esophageal cancer were treated at our hospital, and their medical records were reviewed. The inclusion criteria of this study were as follows: (1) Pathologically confirmed diagnosis of ESCC and excluding other histology type; (2) Patients received preoperative CCRT followed by esophagectomy; (3) Patients had available pretreatment specimens of biopsy for IHC; (4) 7th American Joint Committee on Cancer (AJCC) clinical stage II or III; (5) Eastern Cooperative Oncology Group performance status 0–1. The exclusion criteria of this study were as follows: (1) The presence of distant metastases; (2) Cervical location of esophageal cancer; (3) Patients had synchronous cancer such as head and neck cancer; (4) Patients had a history of malignancy within five years prior to ESCC diagnosis. Finally, 93 patients diagnosed with ESCC receiving preoperative CCRT followed by an esophagectomy were identified. Pretreatment staging included a computed tomography (CT) scan of the chest and abdomen or/and an endoscopic ultrasound (EUS). Patients were evaluated by a multidisciplinary team including a thoracic surgeon, a radiation oncologist, a medical oncologist, a gastroenterologist and a radiologist. The clinical staging was determined according to the 7th American Joint Committee on Cancer (AJCC) staging system (Supplementary Table S1) [25]. Overall survival was computed from the date of diagnosis until death or the last follow-up. Disease-free survival was calculated from the time of the esophagectomy to the diagnosis of recurrent disease or death without evidence of disease recurrence, censoring the date of death.

### 2.2. Treatment Plan

The protocol for preoperative CCRT was described as previously [2]. A post-treatment CT scan was performed to evaluate the treatment response within 3–4 weeks from the end of radiotherapy. The multidisciplinary team reviewed the clinical information to make decisions for an operation or not. If the lesions were classified as resectable, surgery was advised approximately 6–10 weeks from the end of preoperative CCRT. Surgery included a radical esophagectomy with cervical esophagogastrostomy or an Ivor Lewis esophagectomy with intrathoracic anastomosis, two-field lymphadenectomy, reconstruction of the digestive tract with a gastric tube and pylorus drainage procedures. The pCR was defined as the complete disappearance of all viable cancer cells in all surgical specimens including the esophageal lesion and lymph nodes.

### 2.3. Immunohistochemistry (IHC)

The immunoperoxidase technique using 4 μm slides of formalin-fixed, paraffin-embedded tissue sections was performed for IHC staining. In summary, deparaffinization and rehydration were performed first. For antigen retrieval, 10 mM citrate buffer (pH 6.0) for a heat-induced epitope retrieval in a hot water bath (95 °C) was used for 20 min. To block endogenous peroxidase activity, 0.3% hydrogen peroxide was used for 15 min. Then, the slides were put in blocking buffer with 1% goat serum for 1 h at room temperature. Next, we incubated the slides with a galectin-1 primary antibody (HPA000646; Sigma-Aldrich, St. Louis, MO, USA) at a 1:400 dilution for 40 min at room temperature. The LSAB2 kit (Dako, Carpinteria, CA, USA) followed by hematoxylin for counterstaining was used for immunodetection. Human melanoma was used as a positive control [18]. The negative control was an incubation without the primary antibody. The IHC interpretation was carried out independently by two pathologists (S.L.W. and W.T.H) without any clinicopathological information. Galectin-1 staining was graded as no, faint, moderate and strong staining according to the intensity. Both moderate and strong staining intensities were considered positive. The galectin-1 staining was scored as follows: score 0: no or <10% positive staining; score 1: 10% to <40% positive staining; score 2: 40% to <70% positive staining; and score 3: ≥70% positive staining. Both scores 2 and 3 were defined as an overexpression of galectin-1 [18].

### 2.4. Statistical Analysis

The chi-square/Fisher’s exact tests were used to compare the proportion between the two groups. A multivariate analysis of pCR was calculated by logistic regression. The Kaplan–Meier method was used for the univariate survival analysis, and the statistical difference between different groups was tested by a log-rank test. The multivariate survival analysis was computed by a Cox regression model with a stepwise forward fashion to analyze the relative prognostic significance of the parameters. For all analyses, two-sided tests of significance were used with *p* < 0.05 being considered significant. A statistical analysis was performed using the IBM SPSS Statistics, version 17.0 (IBM Corp., Armonk, NY, USA) software package.

## 3. Results

### 3.1. Patient Characteristics

The patient characteristics are shown in Table 1. The median periods of follow-up were 86 months (range, 63.5–143.9 months) for the 20 survivors. The five-year overall and disease-free survival rates were 28% and 20%, respectively. Among these 93 patients, 23 (25%) patients achieved pCR after preoperative CCRT. The five-year overall and disease-free survival rates were 57% and 48% in patients with pCR, respectively. The corresponding rates were 19% and 11% in patients without pCR.

### 3.2. Correlation between Clinicopathological Parameters and the Expression of Galectin-1

**Figure 1:**
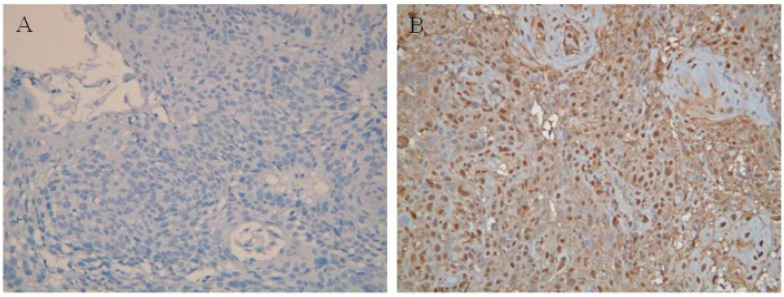
Immunohistochemical staining of galectin-1. (**A**) Representative example of low galectin-1 expression in esophageal squamous cell carcinoma. (**B**) Representative example of galectin-1 overexpression in esophageal squamous cell carcinoma. Original magnification, 200×.

The correlation between clinicopathological parameters and the expression of galectin-1 is summarized in Table 2. Among the 93 patients collected, 46 patients (49%) showed “overexpression” for galectin-1 expression, and the others showed “low expression” (Figure 1). We did not observe any association between galectin-1 expression and any clinicopathologic parameters including age, primary tumor location, histological grade, AJCC 7th staging, T classification and N classification.

### 3.3. Correlation between Clinicopathological Parameters and Pathological Complete Response

The relationship between the clinicopathological parameters and the response of chemoradiotherapy is summarized in Table 3. Low galectin-1 expression (*p* = 0.01) was significantly associated with pCR after preoperative chemoradiotherapy. The clinical 7th AJCC stage (stage II versus stage III) reached a trend (*p* = 0.065). The logistic model showed that low galectin-1 expression (*p* = 0.013, hazard ratio: 3.778, 95% confidence interval: 1.330–10.773) was independently correlated with pCR after chemoradiotherapy.

### 3.4. Survival Analyses

**Figure 2:**
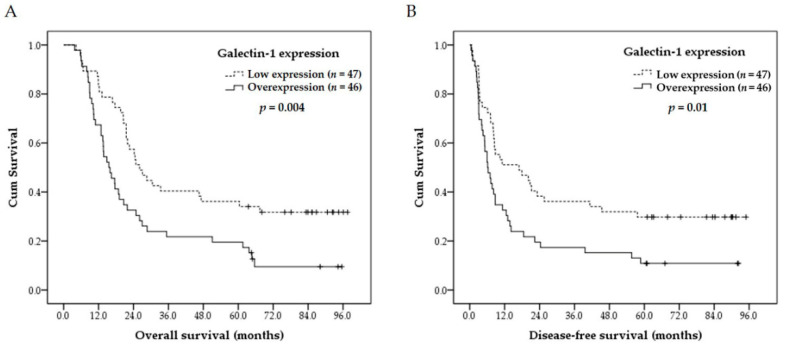
Kaplan–Meier plots to predict (**A**) overall survival and (**B**) disease-free survival according to galectin-1 immunoexpression. Esophageal squamous cell carcinoma patients with galectin-1 overexpression have significantly shorter overall survival and disease-free survival than those with low galectin-1 expression.

Correlations of the clinicopathological parameters and galectin-1 expression with overall survival and disease-free survival are summarized in Table 4. Univariate analyses demonstrated that galectin-1 overexpression (*p* = 0.004, Figure 2A), AJCC 7th stage III (*p* = 0.036) and a positive surgical margin (*p* = 0.001) were significantly associated with inferior overall survival. Additionally, galectin-1 overexpression (*p* = 0.01, Figure 2B), AJCC 7th stage III (*p* = 0.018), clinical N classification, N2/N3 (*p* = 0.038) and a positive surgical margin (*p* = 0.001) were significantly correlated with worse disease-free survival. In multivariate analyses, galectin-1 overexpression (*p* = 0.002, hazard ratio: 2.098, 95% confidence interval: 1.310–3.359, Table 5) and a positive surgical margin (*p* = 0.001, hazard ratio: 2.778, 95% confidence interval: 1.555–4.950, Table 5) represented independent adverse prognosticators for inferior overall survival. Besides, galectin-1 overexpression (*p* = 0.01, hazard ratio: 1.839, 95% confidence interval: 1.157–2.923, Table 5) and a positive surgical margin (*p* = 0.001, hazard ratio: 2.577, 95% confidence interval: 1.462–4.545, Table 5) were also independent adverse prognosticators for worse disease-free survival. The five-year overall and disease-free survival rates were 20% and 11% in patients with galectin-1 overexpression, and 36% and 30% in patients with low galectin-1 expression.

## 4. Discussion

The majority of patients with ESCC have advanced disease when they are diagnosed. The outcome of patients with locally advanced ESCC treated with preoperative CCRT is unsatisfactory, with a five-year survival of around 30% [5,6,7].

To the best of our knowledge, the present study is the first report that galectin-1 is an independent prognosticator in locally advanced ESCC following preoperative CCRT. Our findings suggest that galectin-1 may be a significant biomarker for patients with ESCC treated with preoperative CCRT. Previous several studies have revealed that galectin-1 is associated with poor prognosis in numerous human cancers. Saussez et al. [26] reported that galectin-1 overexpression is a negative prognosticator in patients with laryngeal squamous cell carcinoma. Wu et al. [27] found that galectin-1 overexpression was associated with poor prognosis in patients with hepatocellular carcinoma following resection. In patients with clear cell renal cell carcinoma, White et al. [15] also showed that galectin-1 overexpression was correlated with shorter disease-free survival. However, the significance of galectin-1 expression on the prognosis of patients with locally advanced ESCC treated with preoperative CCRT remains unknown. Thus, we focused on these ESCC patients and determined the significance of galectin-1 expression. Interestingly, we found that galectin-1 overexpression was significantly correlated with poor overall and disease-free survival in the univariate analysis and that it remained independent in the multivariate analysis.

In the present study, we found that there was a significant association between galectin-1 expression and CCRT response, which may partially explain the poor overall and disease-free survival in patients with galectin-1 overexpression. Patients with galectin-1 overexpression had a lower pCR rate than those with low galectin-1 expression. Among 47 patients with low galectin-1 expression, 17 (36%) patients had pCR after preoperative CCRT. However, only six (13%) of 46 patients with galectin-1 overexpression got pCR. Recently, for patients with locally advanced ESCC, treatment modalities of definitive or preoperative CCRT followed by surgery have been advocated in many medical centers [2]. After CCRT, no viable tumors are observed in 20~40% of resected esophageal tumor specimens, and it is associated with therapeutic long-term survival benefits [5,6,7]. On the other hand, preoperative CCRT increases the perioperative morbidity and mortality [28]. The ability to identify patients whose tumor responds to CCRT could allow for a more appropriate decision for multimodality treatment options. Our results indicate that for locally advanced ESCC patients with galectin-1 overexpression, esophagectomy is suggested after chemoradiotherapy if the disease is operable. Our results showed that galectin-1 overexpression was associated with a poor response to CCRT in patients with ESCC. With regard to radiotherapy, Huang et al. [18,29] reported that galectin-1 was an independent prognostic factor for local recurrence and survival after definitive radiotherapy for patients with squamous cell carcinoma of the uterine cervix and that galectin-1 may mediate radioresistance through the H-Ras-dependent pathway involved in DNA damage repair. Chou et al. [21] also found that galectin-1 was a poor prognostic factor in patients with glioblastoma multiforme receiving radiotherapy. Kuo et al. [24] reported that galectin-1 could mediate radiation-related lymphopenia and attenuate the radiation response of non-small cell lung cancer. As for chemotherapy, cisplatin plus 5-FU was the most used regimen during CCRT in our study. Chung et al. [23] revealed that galectin-1 promoted the chemotherapy resistance of cisplatin in lung cancer by upregulating p38 mitogen-activated protein kinase, extracellular signal-regulated kinase and cyclooxygenase-2. Su et al. [19] found that galectin-1-induced autophagy facilitated cisplatin resistance in hepatocellular carcinoma. Zhang et al. [30] showed that lncRNA small nucleolar RNA host gene 22 contributed to the chemotherapy resistance of cisplatin and paclitaxel through the miR-2467/galectin-1 signaling pathway in epithelium ovarian carcinoma. Gao et al. [31] reported that galectin-1 knockdown enhanced the cisplatin sensitivity of neuroblastoma cells by inhibiting autophagy. The above findings suggest that galectin-1 plays an important role in the resistance to radiotherapy and chemotherapy. Therefore, more studies are encouraged to further elucidate the role of galectin-1 in the resistance to radiotherapy and chemotherapy.

For patients with locally advanced ESCC receiving preoperative CCRT, 10–20% patients cannot receive planned esophagectomy due to the development of distant metastases, severe toxicity of preoperative CCRT, patient choice or poor performance status after preoperative CCRT [3,4]. Most of them did not receive the planned esophagectomy due to the development of distant metastases during preoperative CCRT. In the present study, we excluded ESCC patients without a planned esophagectomy after preoperative CCRT. We only enrolled ESCC patients receiving preoperative CCRT followed by a planned esophagectomy because we wanted to evaluate whether galectin-1 expression could predict the response of CCRT, and we suggested that pCR may be the best and clear-cut surrogate marker to represent the response of CCRT. For patients developing distant metastases during preoperative CCRT, their primary tumors may get a good response to CCRT, which highlights the importance of chemotherapy resistance instead of radiotherapy resistance.

Our study has some limitations. First, this was a retrospective study. Second, our observations were limited by the relatively small number of patients.

## 5. Conclusions

Our study showed that galectin-1 overexpression is independently associated with a poor response and worse prognosis in patients with ESCC who were treated with preoperative chemoradiotherapy. Therefore, galectin-1 might be a potential target for therapeutic intervention in ESCC patients who are treated with multimodalities.

## Figures and Tables

**Table 1:** Clinicopathologic features of patients (*n* = 93).

Parameters	No. of Cases (Percentage)
Age (years) (mean: 53.2, median: 52, range: 37–77)	
<50	35 (38%)
50 ≤ Age < 60	35 (38%)
60 ≤ Age < 70	19 (20%)
70 ≤ Age	4 (4%)
Sex	
Male	91 (98%)
Female	2 (2%)
Clinical T classification	
T2	9 (10%)
T3	41 (44%)
T4	43 (46%)
Clinical N classification	
N0	23 (25%)
N1	30 (32%)
N2	29 (31%)
N3	11 (11%)
Clinical 7th AJCC stage	
II	23 (25%)
III	70 (75%)
Tumor grade	
1	16 (17%)
2	54 (58%)
3	23 (25%)
Primary tumor location	
Upper	13 (14%)
Middle	46 (49%)
Lower	34 (37%)
Galectin-1 expression	
Low expression	47 (51%)
Overexpression	46 (49%)
Surgical margin	
Negative	77 (83%)
Positive	16 (17%)
pCR	
Absent	70 (75%)
Present	23 (25%)

**Table 2:** Associations between galectin-1 expression and clinicopathologic parameters.

Parameters	Category	Galectin-1 Expression		
		Low Expression	Overexpression	*p* Value
Age	<52y/o	28	22	0.35
	≥52y/o	29	24	
Clinical T classification	T2/3	29	21	0.12
	T4	18	25	
Clinical N classification	N0	14	9	0.25
	N1/2/3	33	37	
Clinical N classification	N0/1	29	24	0.35
	N2/3	18	22	
Clinical 7th AJCC stage	II	14	9	0.25
	III	33	37	
Tumor grade	1/2	35	35	0.86
	3	12	11	
Primary tumor location	Upper/Middle	27	32	0.23
	Lower	20	14	

**Table 3:** Associations between the pathological complete response and clinicopathologic parameters.

Parameters	Category	Pathological Complete Response		
		Present	Absent	*p* Value
Age	<52y/o	7	33	0.16
	≥52y/o	16	37	
Clinical T classification	T2/3	15	35	0.20
	T4	8	35	
Clinical N classification	N0	8	15	0.20
	N1/2/3	15	55	
Clinical N classification	N0/1	16	37	0.16
	N2/3	7	33	
Clinical 7th AJCC stage	II	9	14	0.065
	III	14	56	
Tumor grade	1/2	18	52	0.70
	3	5	18	
Primary tumor location	Upper/Middle	12	47	0.20
	Lower	11	23	
Galectin-1	Low expression	17	30	0.01 *
	Overexpression	6	40	

* Statistically significant. x^2^ test was used for the statistical analysis.

**Table 4:** Results of the univariate analysis of the prognostic factors for overall survival and disease-free survival.

Factors	No. of Patients	Overall Survival (OS)		Disease-Free Survival (DFS)	
		5-year OS Rate (%)	*p* Value	5-year DFS Rate (%)	*p* Value
Age					
<52y/o	40	25%	0.93	20%	0.99
≥52y/o	53	30%		21%	
Clinical T classification					
T2/3	50	34%	0.13	24%	0.078
T4	43	21%		16%	
Clinical N classification					
N0	23	44%	0.22	30%	0.12
N1/2/3	70	23%		17%	
Clinical N classification					
N0/1	53	36%	0.093	25%	0.038 *
N2/3	40	18%		15%	
Clinical 7th AJCC stage					
II	23	52%	0.036 *	35%	0.018 *
III	70	20%		16%	
Tumor grade					
1/2	70	29%	0.49	21%	0.75
3	23	26%		17%	
Primary tumor location					
Upper/Middle	59	29%	0.83	20%	0.67
Lower	34	27%		21%	
Surgical margin					
Negative	77	34%	0.001 *	25%	0.001 *
Positive	16	0%		0%	
Galectin-1					
Low expression	47	36%	0.004 *	30%	0.01 *
Overexpression	46	20%		11%	

* Statistically significant.

**Table 5:** Results of the multivariate Cox regression analysis for overall survival and disease-free survival in 93 patients with locally advanced esophageal squamous cell carcinoma receiving preoperative chemoradiotherapy.

Factors	Overall Survival		Disease-free Survival	
	HR (95% CI)	*p* Value	HR (95% CI)	*p* Value
Galectin-1 overexpression	2.098 (1.310–3.359)	0.002 *	1.839 (1.157–2.923)	0.01 *
Positive surgical margin	2.778 (1.555–4.950)	0.001 *	2.577 (1.462–4.545)	0.001 *

HR, hazard ratio; 95% CI, 95% confidence interval; * Statistically significant.

## Data Availability

The data presented in this study are available upon request from the corresponding author. The data are not publicly available due to ethical restrictions.

## Supplementary Materials

The following are available online at https://www.mdpi.com/article/10.3390/cancers13133147/s1, Table S1: American Joint Committee on Cancer (AJCC) TNM Classification of Carcinoma of the Esophagus and Esophagogastric Junction (7th ed, 2010)

## Abbreviations

Esophageal squamous cell carcinoma	ESCC
Concurrent chemoradiotherapy	CCRT
Pathological complete response	pCR
Immunohistochemistry	IHC
American Joint Committee on Cancer	AJCC
Computed tomography	CT
Endoscopic ultrasound	EUS
