# Supplementary material for: Galectin-1 Expression Is Associated with the Response and Survival Following Preoperative Chemoradiotherapy in Locally Advanced Esophageal Squamous Cell Carcinoma
Source: Cancers (Basel). 2021 Jun 23;13(13):3147. doi: 10.3390/cancers13133147 (PMC8268777; doi:10.3390/cancers13133147)
Supplement: Supplementary file 1 [file cancers-13-03147-s001.zip › cancers-1226994-supplementary.pdf]

Supplementary Table S1. American Joint Committee on Cancer (AJCC) TNM Classification of Carcinoma of the Esophagus and Esophagogastric Junction (7th ed, 2010)

**Primary Tumor (T)**	
Tx	Primary tumor cannot be assessed
T0	No evidence of primary tumor
Tis	High-grade dysplasia*
T1	Tumor invades lamina propria, muscularis mucosae, or submucosa
T1a	Tumor invades lamina propria or muscularis mucosae
T1b	Tumor invades submucosa
T2	Tumor invades muscularis propria
T3	Tumor invades adventitia
T4	Tumor invades adjacent structures
T4a	Resectable tumor invading pleura, pericardium, or diaphragm
T4b	Unresectable tumor invading other adjacent structures, such as aorta, vertebral body, trachea, etc.

*High-grade dysplasia (HGD) includes all non-invasive neoplastic epithelium that was formerly called carcinoma in situ, a diagnosis that is no longer used for columnar mucosae anywhere in the gastrointestinal tract.

**Regional Lymph Nodes (N)**	
NX	Regional lymph nodes cannot be assessed
N0	No regional lymph node metastasis
N1	Metastases in 1 - 2 regional lymph nodes
N2	Metastases in 3 - 6 regional lymph nodes
N3	Metastases in 7 or more regional lymph nodes

**Distant Metastasis (M)**	
M0	No distant metastasis
M1	Distant metastasis

Anatomic Stage/Prognostic Groups

Squamous cell carcinoma*

Stage	T	N	M	Grade	Tumor location**
Stage 0	Tis (HGD)	N0	M0	1, X	Any
Stage IA	T1	N0	M0	1, X	Any

Stage IB	T1	N0	M0	2-3	Any
	T2-3	N0	M0	1, X	Lower, X
Stage IIA	T2-3	N0	M0	1, X	Upper, middle
	T2-3	N0	M0	2-3	Lower, X
Stage IIB	T2-3	N0	M0	2-3	Upper, middle
	T1-2	N1	M0	Any	Any
Stage IIIA	T1-2	N2	M0	Any	Any
	T3	N1	M0	Any	Any
	T4a	N0	M0	Any	Any
Stage IIIB	T3	N2	M0	Any	Any
Stage IIIC	T4a	N1-2	M0	Any	Any
	T4b	Any	M0	Any	Any
	Any	N3	M0	Any	Any
Stage IV	Any	Any	M1	Any	Any

*Or mixed histology including a squamous component or NOS.
**Location of the primary cancer site is defined by the position of the upper (proximal) edge of the tumor in the esophagus.

Anatomic Stage/Prognostic Groups

Adenocarcinoma

Stage	T	N	M	Grade
Stage 0	Tis (HGD)	N0	M0	1, X
Stage IA	T1	N0	M0	1-2, X
Stage IB	T1	N0	M0	3
	T2	N0	M0	1-2, X
Stage IIA	T2	N0	M0	3
Stage IIB	T3	N0	M0	Any
	T1-2	N1	M0	Any
Stage IIIA	T1-2	N2	M0	Any
	T3	N1	M0	Any
	T4a	N0	M0	Any
Stage IIIB	T3	N2	M0	Any
Stage IIIC	T4a	N1-2	M0	Any
	T4b	Any	M0	Any
	Any	N3	M0	Any

Stage IV	Any	Any	M1	Any

Histologic Grade(G)

GX	Grade cannot be assessed-stage grouping as G1
G1	Well differentiated
G2	Moderately differentiated
G3	Poorly differentiated
G4	Undifferentiated-stage grouping as G3 squamous
